# Identification and Classification of Novel Genetic Variants: En Route to the Diagnosis of Primary Ciliary Dyskinesia

**DOI:** 10.3390/ijms22168821

**Published:** 2021-08-17

**Authors:** Nina Stevanovic, Anita Skakic, Predrag Minic, Aleksandar Sovtic, Maja Stojiljkovic, Sonja Pavlovic, Marina Andjelkovic

**Affiliations:** 1 Laboratory for Molecular Biomedicine, Institute of Molecular Genetics and Genetic Engineering, University of Belgrade, 11010 Belgrade, Serbia; nina.stevanovic@imgge.bg.ac.rs (N.S.); skakic.anita@gmail.com (A.S.); maja.stojiljkovic@yahoo.com (M.S.); sonya@sezampro.rs (S.P.); 2 Mother and Child Health Care Institute of Serbia Dr. VukanCupic, 11070 Belgrade, Serbia; pbminic@gmail.com (P.M.); adsovtic@gmail.com (A.S.); 3 School of Medicine, University of Belgrade, 11000 Belgrade, Serbia

**Keywords:** PCD, NGS, *DNAI1*, *SPAG16*, functional analysis, in silico structural analysis

## Abstract

Primary ciliary dyskinesia (PCD) is a disease caused by impaired function of motile cilia. PCD mainly affects the lungs and reproductive organs. Inheritance is autosomal recessive and X-linked. PCD patients have diverse clinical manifestations, thus making the establishment of proper diagnosis challenging. The utility of next-generation sequencing (NGS) technology for diagnostic purposes allows for better understanding of the PCD genetic background. However, identification of specific disease-causing variants is difficult. The main aim of this study was to create a unique guideline that will enable the standardization of the assessment of novel genetic variants within PCD-associated genes. The designed pipeline consists of three main steps: (1) sequencing, detection, and identification of genes/variants; (2) classification of variants according to their effect; and (3) variant characterization using in silico structural and functional analysis. The pipeline was validated through the analysis of the variants detected in a well-known PCD disease-causing gene (*DNAI1*) and the novel candidate gene (*SPAG16*). The application of this pipeline resulted in identification of potential disease-causing variants, as well as validation of the variants pathogenicity, through their analysis on transcriptional, translational, and posttranslational levels. The application of this pipeline leads to the confirmation of PCD diagnosis and enables a shift from candidate to PCD disease-causing gene.

## 1. Introduction

Primary ciliary dyskinesia (PCD(OMIM #244400)) is a rare genetic disorder most often inherited in an autosomal recessive and X-linked manner; however, recent studies have shown that autosomal dominant variants in the *FOXJ1* gene also cause PCD [1]. The main features of PCD are structural defects of motile cilia, leading to abnormal ciliary motility, ciliary immotility, or absence of cilia [2]. Because motile cilia are present in humans in the respiratory tract, middle ear, paranasal sinuses, female reproductive tract, ependyma of the brain, and on the embryonic node, this disorder predominantly affects the respiratory system, reproductive organs, and visceral organ laterality (about 50% of the PCD patients have situs inversus) [3]. PCD patients struggle with recurrent and chronic upper and lower respiratory tract infections; productive cough; rhinitis; recurrent otitis media [4,5] bronchiectasis; and, in some cases, progressively declining lung function [6] due to impaired mucociliary clearance (MCC) [7]. Manifestations in other systems include subfertility in both males, due to immotile spermatozoa [8], and females, due to altered motility of Fallopian tube cilia [9]. The prevalence of the disease is very variable across Europe, and it is often underdiagnosed due to the inaccessibility of diagnostic facilities [10]. The estimated prevalence is between 1:4000 and 1:40,000, with the true prevalence of probably about 1:10,000 [5,10] and higher rates in certain ethnic groups due to consanguinity [11,12].

The clinical manifestations of PCD are well known and described in the scientific literature [7,13], but establishing an accurate diagnosis on time is still a challenge. Many diagnostic tests and procedures, such as nasal nitric oxide measurement, saccharin test, and nasal mucociliary transport tests, are not available to all physicians in different countries; moreover, some of the symptoms, are similar to symptoms of other lung diseases, especially in small children. Nowadays, the understanding of the differences in respiratory disease severity has been improved as a result of accumulated knowledge of the genetic basis of PCD [14].

Motile ciliopathies are likely a set of disorders, such that some biallelic pathogenic variants may not cause typical PCD presentation but could result in moderately reduced function of motile cilia and less severe clinical manifestations [15,16]. Indeed, scientific data have suggested that different genetic variants in the same PCD disease-causing gene result in different disease severity [15,16].

At present, about 40 genes have been associated with the disease, and more than 70% of patients have been shown to be carriers of two pathogenic genetic variants (in homozygous or compound heterozygous state) in one of these genes. That number will increase with further genetic screening, thanks to next-generation sequencing (NGS)technologies. The application of targeted exome sequencing (TES) and whole-exome sequencing (WES) to identify variants that cause PCD has resulted in the identification of novel candidate genes [4,11,17,18,19,20,21,22]. However, identification of the specific disease-causing genetic variants is very difficult due to a large amount of data of unknown significance for the disease generated by using these methods.

The main aim of this work was to optimize the analysis of a large number of genetic variants, declared as potential disease-causing variants, to assess their actual pathogenic and clinical impact. Herein, we designed a pipeline to detect and classify the rare genetic variants detected using NGS, specifically, targeted exome sequencing, which enables us to evaluate all detected variants in the same manner. The pipeline included both in silico and functional analysis of the detected variants. Guided by a designed pipeline, previously detected genetic variants in the *DNAI1* (dynein axonemal intermediate chain 1) gene as well as in the *SPAG16* (sperm-associated antigen 16) gene were analyzed.

## 2. Results

**Figure 1:**
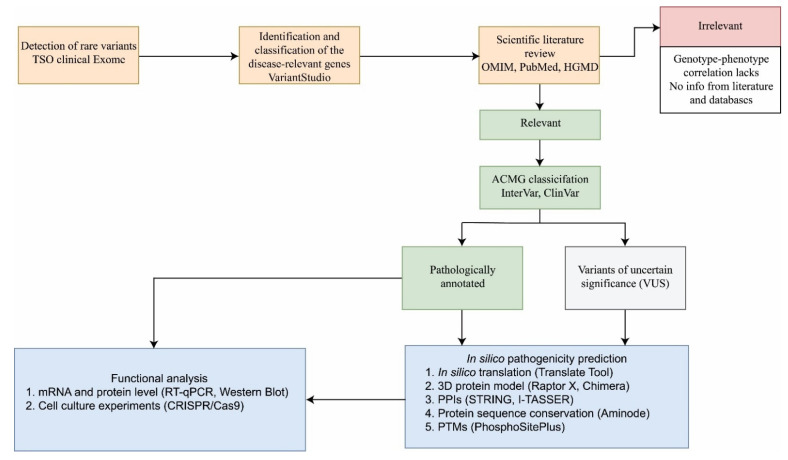
Pipeline designed for identification, classification, and functional analysis of novel genetic variants and candidate genes. The pipeline consists of three main steps. Orange rectangles represent the first step: (1) sequencing, detection, and identification of genes/variants. TruSight One Gene Panel was used for the sequencing of the samples. The Variant Studio software was used for detection and variant filtering. Relevant variants were checked using OMIM, HGMD, and PubMed databases. Irrelevant variants were labeled with the reddish rectangle. The green rectangles represent the second step: (2) classification of variants according to their effect. ACMG classification (within the Varsome database), InterVar software, and ClinVar database were used to classify detected variants according to their consequences. The blue rectangles represent the third step of the pipeline: (3) functional analysis and/or in silico analysis. The variants labeled as pathogenic can be directly functionally characterized but can also be subjected to in silico analysis. For the variants of unknown significance, the in silico pathogenicity prediction precedes the experimental analyses. PPIs—protein-protein interactions; PTMs—posttranslational modifications.

NGS analysis generates a large amount of data, i.e., thousands of genetic variants that need to be carefully categorized. To identify, classify, and characterize novel genetic variants and novel candidate genes using in silico and functional analysis as tools, we designed the guideline in a form of a pipeline (Figure 1).

The pipeline is based on three main steps: (1) sequencing, detection, and identification of genes/variants; (2) classification of variants according to their effect; and (3) functional characterization and/or in silico structural and functional analysis.

### 2.1. Sequencing, Detection, and Identification of Genes/Variants

Variants detected using the Illumina Clinical-Exome Sequencing (CES) TruSight One Gene Panel were analyzed by using Variant Studio Software. All variants that are filtered-in (filters are described in detail in [23]) were assessed in depth by the following databases: OMIM, HGMD, and PubMed. After detailed research of the scientific literature and database-based evidence, if a variant was assessed as irrelevant, it was not further taken into consideration. Irrelevant variants lack genotype–phenotype correlation, and no association of the variant with any structure/function responsible for this disease or with symptoms of the disease was found on the basis of the scientific literature.

### 2.2. Classification of Variants, and Functional Analysis and/or In Silico Analysis

Relevant variants were further processed using the InterVar software tool and ACMG classification, ClinVar, and Varsome databases. The novel variants that were annotated as pathogenic as well as the variants that were annotated as variants with uncertain significance (VUS), according to ACMG classification and ClinVar, were further analyzed with in silico tools and/or the experiments were performed to confirm or to refute the pathogenicity of the variants.

In silico pathogenicity prediction includes a prediction of the impact of the detected variants on transcriptional, translational, and posttranslational levels. When the variant changes the open reading frame (ORF) of the sequence, Translate Tool software [24] was used for the detection of all the possible ORFs. Usage of tools such as Raptor X [25], Phyre2 [26], and Chimera provided the three-dimensional predicted models of selected proteins and information on how the genetic variant impacts the folding of the protein. When the genetic variant disrupts the interaction of the protein with other proteins as well as ligand binding sites, consequently the original protein function may be altered or completely absent. In silico prediction tools such as STRING [27,28] and COACH [29,30] provided necessary information about the quaternary structure of the protein of interest. To establish whether the genetic variant alters posttranslational modifications (PTMs) of the protein, we used the program PhosphoSitePlus [31]. If the region of protein or the amino acid that is affected by the genetic variant is highly evolutionary conserved, there is an indication that the region is important to maintain the structure or function of a protein or domain. Usage of the Aminode software [32] and protein sequence alignment among different species provides valuable information.

The functional analysis includes experiments on mRNA and protein levels. Moreover, gene editing in certain cell lines could contribute to the elucidation of the impact of novel variants. To investigate the potential effect of the variant that is annotated as pathogenic or variant of uncertain significance (missense, small insertions, deletions, premature stop codons) on gene expression level, we performed the RT-qPCR method. To examine the impact of a detected genetic variant on the protein level, we used Western blot analysis. To establish the linkage between genetic variants and biological phenotypes, we used CRISPR/Cas9 technology, the method that provides all necessary information.

### 2.3. Analysis of the Genetic Variants within the DNAI1 Gene According to Designed Pipeline

#### 2.3.1. Sequencing, Detection, and Identification of Genes/Variants

To determine the genetic basis of the suspected PCD patient, labeled as P21 [23], we used Illumina CES and TruSight One Gene Panel. Detected variants (9000) were analyzed by using Variant Studio 3.0 Software. After the elimination of the irrelevant variants, the 112 genetic variants detected in PCD disease-causing genes, PCD-associated genes, genes belonging to the same gene family as PCD genes, and genes coding for proteins that interact with PCD proteins (in-house PCD gene panel) were assessed in-depth by the following databases: OMIM, HGMD, and PubMed.

#### 2.3.2. Classification of Variants According to Their Effects

The 112 genetic variants were detected in the following genes: *CCDC39*, *CCDC40*, *CCDC103*, *DNAI1*, *DNAI2*, *DNAH5*, *DNAH9*, *DNAH11*, *DNAAF1*, *DNAAF2*, *NME8*, *RPGR*, *RSPH4a*, and *HYDIN*.

After analysis of all detected variants using databases ClinVar and Varsome and InterVar software, 110 variants were no further taken into account because they were synonymous, frequent in European populations (≥5%), and/or benign. Two genetic variants that were detected, c.1345_1349delCTTAA (p.Asn450LeufsTer6) and c.1684G > A (p.Asp562Asn) in the DNAI1 gene (NM_012144.2), were further analyzed, and Sanger sequencing confirmed the results gained from Illumina CES and also revealed that the one variant was inherited from each parent [23]. Moreover, genomic profiling has excluded the occurrence of these variants in the healthy control subjects.

Genetic variant c.1345_1349delCTTAA in the *DNAI1* gene based on the ACMG classification was characterized as pathogenic and was assigned the PVS1, PM2, and PP3 criteria. Genetic variant c.1684G > A in the *DNAI1* gene was characterized as a variant of unknown significance on the basis of the ACMG classification. The criteria PM2, PP3, and BP1 were assigned to the mentioned variant. Using the Varsome database, c.1684G > A in the *DNAI1* gene was characterized as damaging on the basis of the DANN score (0.9993). SIFT and Provean tools characterized genetic variant c.1684G > A in the *DNAI1* gene as pathogenic with a score of 0.00 (SIFT), and the score of the variant was beyond the threshold of −2.5 (Provean). There was no information in the ClinVar database since they are found in our study for the first time.

#### 2.3.3. Computational Analyses of the DNAI1 Protein

**Figure 2:**
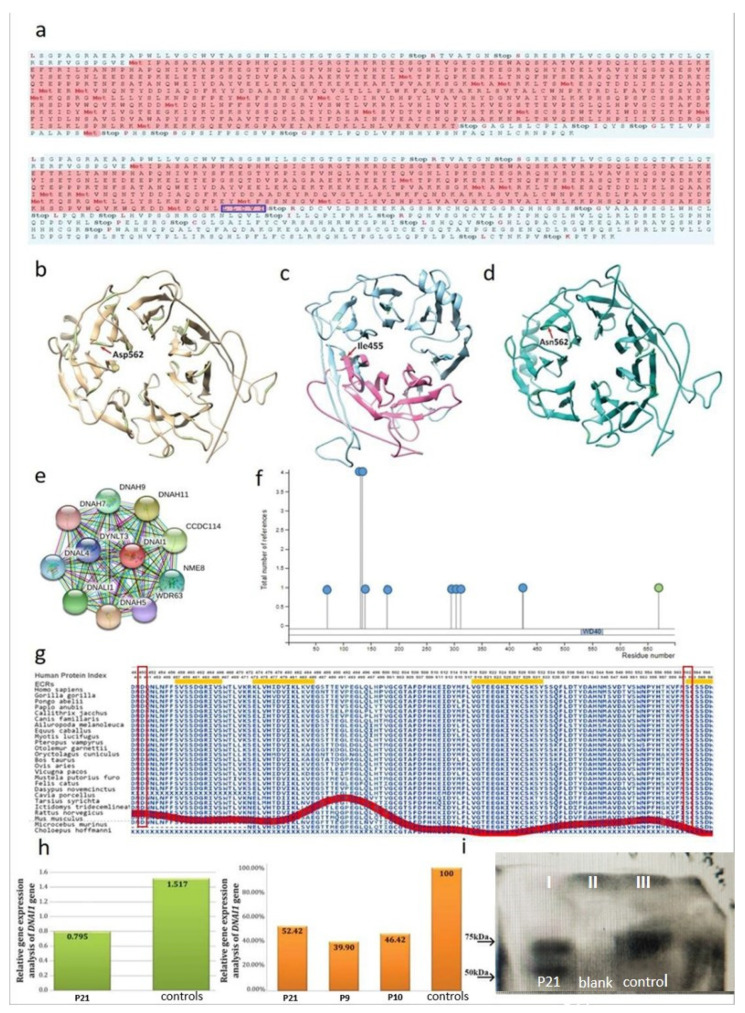
Three-dimensional molecular models of wild-type and mutated DNAI1 (dynein axonemal intermediate chain 1) proteins. In silico and functional analysis of the impact of the detected variants. (**a**) Translation of wild-type (upper figure) and mutated *DNAI1* (lower figure) mRNA sequences. Frameshift mutation p.Asn450LeufsTer6led to a premature stop codon at position 456 in the polypeptide chain. The amino acid substitutions marked with a blue rectangle preceded the stop codon. (**b**) The three-dimensional model of the wild-type DNAI1 protein. (**c**) The three-dimensional model of the DNAI1 protein harboring frameshift mutation, p.Asn450LeufsTer6. The blue color shows the parts of the protein that are missing from the mutated protein, the pink color indicates the part of the protein that is common to wild-type and the mutated protein. (**d**) The three-dimensional model of the DNAI1 protein harboring missense mutation p.Asp562Asn. The codon for aspartic acid (GAC) at position 562 of the polypeptide chain is replaced with the codon for the amino acid asparagine (AAC). (**e**) Multiple protein–protein interactions of the wild-type DNAI1 protein obtained by STRING database. (**f**) Post-translational modifications of wild-type DNAI1 protein gained by use of PhosphoSitePlus program. Blue circles represent phosphorylation, green circles acetylation. (**g**) Amino acid sequence alignment of the DNAI1 protein among different species was determined applying the Aminode platform. The amino acids labeled with a red rectangle displayed complete evolutionary conservation among all analyzed species. Local maxima of the red line indicate protein regions with relatively low evolutionary constrains, while local minima indicate evolutionarily constrained regions (ECRs). Amino acids below the yellow rectangles are highly evolutionary conserved. (**h**) Comparison of the RT-qPCR profile of a child patient labeled as P21, carrying the variants c.1345_1349delCTTAA and c.1684G > A, and control group of children. Relative expression of the *DNAI1* transcript was 50% lower in the affected patient compared to a mean value of healthy controls (left figure). Reduced level of mRNA expression of the *DNAI1* gene presented as a percentage in patients who are carriers of pathogenic variants in the *DNAI1* gene: P21 (c.1345_1349delCTTAA and c.1684G > A), and P9 and P10 (c.947_948insG) relative to the mean expression value of the control group (mean of healthy control samples represents 100% expression) (right figure). (**i**) Using a Western blot method, in a patient sample (P21: p.Asn450LeufsTer6 and p.Asp562Asn), we detected the DNAI1 protein in two different lengths, 699 aa and 455 aa, which corresponds to the wild-type and mutated protein, respectively (lane 1). Lane 2 represents blank. Lane 3 represents positive control for the wild-type DNAI1 protein. The labels in the figure represent the molecular weight of the protein using Precision Plus Protein Dual Color Standards (Bio-Rad, Hercules, CA, USA).

The ExPASy Translate Tool software was used to determine the open reading frame of the wild-type and mutated *DNAI1* gene sequence (Figure 2a). By in silico translation of the wild-type (upper figure) and mutated *DNAI1* gene (lower figure), we detected that the genetic variant c.1345_1349delCTTAA (p.Asn450LeufsTer6) introduces a premature stop codon, which translates a protein that is 244 amino acid (aa) shorter than the wild-type protein.

A three-dimensional model of the wild-type DNAI1 protein was designed using Phyre2 and Chimera software (Figure 2b). The wild-type DNAI1 protein consists of 699 aa, while it was found that the genetic variant c.1345_1349delCTTAA (p.Asn450LeufsTer6) in the *DNAI1* gene, leads to the expression of a 244 aa shorter protein product by in silico analysis (Figure 2c).

The *DNAI1* gene harboring the second detected variant, c.1684G > A (p.Asp562Asn), changes the codon for aspartic acid (GAC) at the position 562 of the polypeptide chain to the codon for the amino acid asparagine (AAC) (Figure 2d).

The STRING database was used for the determination of multiple protein–protein interactions (PPIs) (Figure 2e). According to the database, the DNAI1 protein interacts with 55 different proteins (not all shown), not necessarily physically binding each other, but proteins jointly contribute to a shared function.

By using I-TASSER [33], the consensus ligand-binding residues were predicted at positions: 337, 352, 387, 404, 436, 456, 458, 498, 500, 517, 541, 542, 562, 568, 603, 631, and 633 in polypeptide chain. The binding partner, commonly referred to as ligand, can be metal ions, small organic/inorganic molecules, or macromolecules such as proteins or nucleic acids [34]. In altered protein that harbor p.Asn450LeufsTer6 genetic variant, all the ligand-binding amino acids downstream of the termination codon are missing, and the protein lacks its original quaternary structure. In the mutated protein with p.Asp562Asn genetic variant, the aspartic acid in position 562 in the polypeptide chain of the DNAI1 protein is replaced with amino acid asparagine. Aspartic acid in this position is one of the ligand-binding residues in the wild-type protein. Replacing aspartic acid with asparagine disrupts protein–protein interactions, and likely the function of this protein.

Posttranslational modifications of the DNAI1 protein were analyzed using the program PhosphoSitePlus. A total of 11 posttranslational modifications were detected (phosphorylation and acetylation; Figure 2f). One WD-40 domain was detected (Figure 2f), and the localization of the domain within the DNAI1 protein was in accordance with the predicted region of consensus binding residues. Genetic variants, p.Asn450LeufsTer6, and p.Asp562Asn altered the WD-40 domain, and thus the protein–protein interactions were disabled, leading to loss of the protein function.

Aminode platform was used for amino acid sequence alignment of the DNAI1 protein among different species. Multiple sequences were aligned, and results indicate high evolutionary conservation of residues affected with p.Asn450LeufsTer6 and p.Asp562Asn variants among the 25 orthologs (Figure 2g).

#### 2.3.4. Functional Analysis of the *DNAI1* Gene and Protein

##### Analysis of Expression Levels of the *DNAI1* Gene

The influence of the two genetic variants c.1345_1349delCTTAA (p.Asn450LeufsTer6) and c.1684G > A (p.Asp562Asn) in the *DNAI1* gene on expression level was examined by the qRT-PCR method (Figure 2h). The quantification analysis showed that the expression level of the *DNAI1* gene in a patient was approximately 50% reduced compared to the mean expression level observed in the control group of subjects. The expression level in a child patient was 0.795, while the mean value of expression levels in child controls was 1.517. Further, the relative *DNAI1* gene expression levels of children patients carrying the pathogenic variants introducing a premature stop codon in the DNAI1 gene (P21: c.1345_1349delCTTAA and c.1684G > A, and P9 and P10: c.947_948insG) were compared, and it was shown that genetic variants that alter the open reading frame reduce the expression level of this gene.

##### Immunoblotting of the DNAI1 Protein

The protein transcribed from the *DNAI1* gene was analyzed by the Western blot method in order to determine the influence of the genetic variants c.1345_1349delCTTAA (p.Asn450LeufsTer6) and c.1684G > A (p.Asp562Asn) on the length and amount of protein product. In the analyzed patient sample, a wild-type protein length of 699 amino acids and a molecular weight of 70 kD were detected, as well as a shorter protein length of 455 amino acids (molecular weight of around 55 kD). In the patient, the amount of DNAI1 protein of length 699 amino acid is less than the amount of wild-type protein detected in the control sample (Figure 2i).

### 2.4. Analysis of the Genetic Variant within SPAG16 Gene According to Designed Pipeline

The homozygous genetic variant within the *SPAG16* gene, c.1067G > A, was previously detected in our study in a patient with PCD [23]. Although this gene had never been associated with PCD before, the genotype–phenotype correlation was immediately established, and *SPAG16* was declared as a candidate gene for PCD. Within this study, using a designed pipeline, we performed a very detailed in silico analysis of the variant c.1067G > A in the *SPAG16* gene in order to confirm the effects on protein.

The first two steps of the pipeline were accomplished in our previous study [23], and thus the variant c.1067G > A in the *SPAG16* gene was further proceeded to the third step.

#### Computational Analyses of the SPAG16 Protein

**Figure 3:**
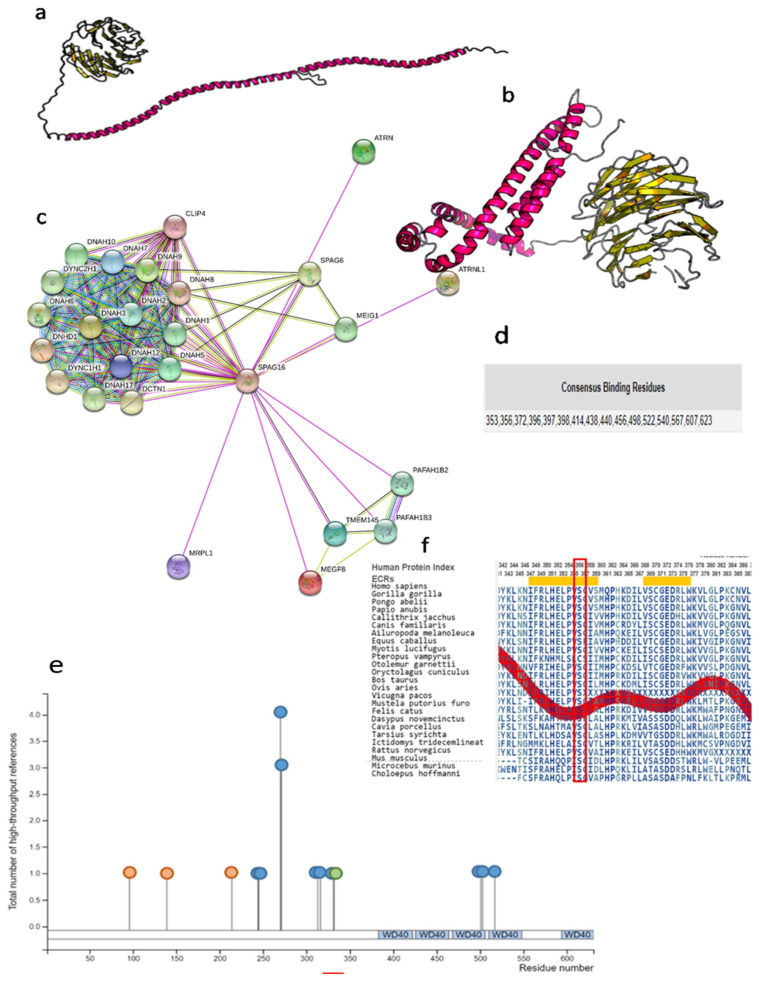
Three-dimensional molecular models of wild-type and mutated SPAG16 (sperm-associated antigen 16) protein. In silico confirmation of the impact of the detected variant on protein level. (**a**) The three-dimensional model of the wild-type SPAG16 protein. (**b**) The three-dimensional model of the SPAG16 protein harboring amino acid change, p.Ser356Asn. Both proteins are the same length, but due to the genetic variant in the *SPAG16* gene, the tertiary structure of the mutated protein differs from the original tertiary structure of wild-type protein. (**c**) Multiple protein–protein interactions of the wild-type SPAG16 protein. These results were obtained applying the STRING database. (**d**) Consensus binding residues of the wild-type SPAG16 protein. Among others, amino acid serine in position 356 in the polypeptide chain of the wild-type protein is involved in PPIs. In the mutated SPAG16 protein harboring the p.Ser356Asn variant, the amino acid serine is replaced with amino acid asparagine in position 356, which may affect the interactions with ligands. COACH server was used for gaining these results. (**e**) Post-translational modifications of the wild-type SPAG16 protein obtained by the PosphoSitePlus program. Blue circles represent phosphorylation, green circles acetylation, and orange circles represent ubiquitination. (**f**) Amino acid sequence alignment of the SPAG16 protein, among different species. The amino acids labeled with a red rectangle displayed complete evolutionary conservation among all analyzed species. Local maxima of the red line indicate protein regions with relatively low evolutionary constrains, while local minima indicate evolutionarily constrained regions (ECRs). Amino acids below the yellow rectangles are highly evolutionary conserved. Amino acid sequence alignment was performed with the Aminode.

Using Raptor X software, we produced three-dimensional predicted models of wild-type and mutated SPAG16 proteins, as shown in Figure 3a,b. Both proteins, the wild-type and the one harboring one amino acid change p.Ser356Asn, consist of 631 amino acids. According to the predicted models of unchanged and mutated proteins, the tertiary structure of these two proteins significantly differs. Although the sequence similarity is almost 100%, the amino acid change in the primary structure of mutated SPAG16 protein consequently alters the secondary structure of the protein, and the effect of this amino acid substitution is the most evident on the tertiary structure of this protein. Further, the altered tertiary structure affects the protein–ligand interaction due to the inaccessibility of certain parts of the protein for PPIs.

Multiple protein–protein interactions were detected using the STRING database, and the predicted quaternary structure of the wild-type SPAG16 protein is shown in Figure 3c. Among others, the SPAG16 protein directly interacts with SPAG6 protein, as well as with numerous ciliary proteins. By using meta-server approach, COACH, the consensus ligand-binding residues were predicted at positions 353, 356, 372, 396, 397, 398, 414, 438, 440, 456, 498, 522, 540, 567, 607, and 623 in polypeptide chain (Figure 3d). Amino acid serine in 356 position in the polypeptide chain of the SPAG16 protein is one of the ligand-binding residues in the wild-type protein. In the mutated protein, this amino acid is replaced with amino acid asparagine which may affect the PPIs.

As PTMs significantly increase proteome functionality, posttranslational modifications of SPAG16 protein were analyzed using the program PhosphoSitePlus. A total of 14 modifications were detected by type phosphorylation, acetylation, and ubiquitination (Figure 3e). Five WD-40 domains were detected (Figure 3e). Localization of the WD-40 domains within the SPAG16 protein was in accordance with the predicted region of consensus binding residues gained by the COACH server.

Amino acid sequence alignment among different species was performed using the Aminode platform. Multiple sequences were aligned, and results indicate high evolutionary conservation of residues affected with p.Ser356Asn variant, among the SPAG16 orthologs in all analyzed species (Figure 3f).

## 3. Discussion

To establish an accurate diagnosis of PCD, in addition to the analysis of known variants within the disease-causative genes, it is necessary to analyze other pathogenic variants, as well as potential candidate genes for the disease. In the present study, we report a very comprehensive genetic pipeline designed for identification, classification, and structural and functional analysis of novel genetic variants and candidate genes. In addition to the biallelic genetic variants that are included in most of the previously designed pipelines known from scientific papers [35,36], this guideline enables the analysis of the variants with unknown significance that are detected very often when the NGS method is applied. Further, this guideline enables the analysis of the candidate genes for PCD, which are rapidly increasing, given the number of genes and proteins that participate in the proper structure and functioning of motile cilia. The advantage of this approach is the possibility of gene shift from candidate to PCD disease-causing gene. Further, the variants labeled as VUS could be analyzed so they could gain diagnostic significance rather than they were ruled out due to their diagnostic unclarity. However, there are limitations of the pipeline designed in this way. This pipeline is based on genetic aspects of the disease, and it is useful to confirm a genetic diagnose of already clinically suspected PCD patients. The biggest limitation of this pipeline compared to the already existing pipelines [35,36] is the lack of other methods (such as imaging) that could confirm ciliary abnormality even when biallelic genetic variants were not detected.

This pipeline consists of three main steps, and its validation was performed through the analysis of one PCD disease-causing gene, the *DNAI1* gene, and one candidate gene for PCD, the *SPAG16* gene.

The first step of the pipeline incorporated sequencing, detection, and identification of genes or genetic variants. The genetic panel that we used contains the 29 disease-causing and candidate genes for PCD [23]. The limitation of this panel is an incomplete list of the known PCD genes (OMIM), which, in some rare cases, may lead to a diagnosis failure. However, the utilization of NGS in comparison to targeted Sanger sequencing of individual genetic variants leads to a better mutation detection rate [23,37] and should be used as a routine diagnostic test for PCD patients. Phenotype-driven gene lists were generated on the basis of a search of OMIM and HGMD databases, as well as PubMed, and the second step of the pipeline was further applied to the PCD relevant genes. These databases are commonly used in various studies in which the WES and CES were applied [38,39,40].

Guided by the first step of the pipeline for analysis of PCD genetic variants, we detected a total of three genetic variants, two harboring the PCD disease-causing gene, the *DNAI1*, and one in the homozygous state within the *SPAG16* gene, the novel candidate gene for PCD. The *DNAI1* gene is the first gene associated with the development of PCD [41]. Genetic variants present in the *DNAI1* gene are associated with the absence of ODA, as well as altered and reduced ciliary movement, and have been detected in more than 9% of all PCD patients [42,43]. The genetic variant c.1345_1349delCTTAA (p.Asn450LeufsTer6) in the *DNAI1* gene leads to an alteration in the open reading frame and the generation of a premature stop codon. The genetic variant c.1684G > A (p.Asp562Asn) in the *DNAI1* gene introduces asparagine in place of aspartic acid in the polypeptide chain of the DNAI1 protein. These pathogenic genetic variants detected in the *DNAI1* gene have not been described in the literature thus far.

In our previous study [23], for the first time, the *SPAG16* gene was enrolled as a novel candidate gene for PCD. Homozygous pathogenic genetic variant in the *SPAG16* (c.1067G > A) gene was detected for the first time in our study in a male patient with ciliary dyskinesia, slowly motile airway cilia, bronchiectasis, and sinusitis. Previously, this gene, as well as other *SPAG* family members, were associated with some symptoms of the disease, but never with PCD [44,45]. The SPAG16 protein is a part of a bridge-like structure connecting two central microtubules of cilia [46]. It is associated with the axonemal central apparatus of cilia and flagella that is essential for ciliary and flagellar motility in mammals. Recent data derived from high-throughput studies revealed expression of the *SPAG16* gene in multi-ciliated and non-ciliated tissues and have shown that the expression of *SPAG16* mRNA was higher in the tissues bearing motile cilia and testis than in the other analyzed tissues [47]. Furthermore, experiments with double mice knockouts of *Spag6* and *Spag16* are infertile but also developed lung pathologies [48,49]. These results implying the importance of the *SPAG16* gene in ciliary and flagellar motility clearly suggest the association between the *SPAG16* gene and PCD.

Detected variants were further classified using ACMG classification [50], and genetic variants labeled as pathogenic, as well as variants with uncertain significance, were proceeded to the third step.

Detected genetic variants within the *DNAI1* gene, c.1345_1349delCTTAA and c.1684G > A, were classified as pathogenic and the variant of uncertain significance, respectively. The variant, c.1067G > A detected in the *SPAG16* gene, was classified as a variant of uncertain significance. In addition to its unquestionable clinical utility, NGS is also unveiling a large amount of VUS, which we are still not able to precisely define and classify [51]. Further analysis of a VUS by in silico structural analysis and functional characterization, as well as the standardization of their interpretation, are crucial for making VUS qualified when making a final genetic diagnosis of PCD.

The in silico structural and/or functional analysis is the last step of the designed pipeline, and only a few selected variants were subjected to these analyses. If the DNA and mRNA sequence of the gene of interest is modified due to the presence of a genetic variant, the biological function of the translated protein is altered since the biological function of a protein is dependent on its native 3D structure. The techniques of structure determination, such as XRD or NMR [52], are highly expensive, and therefore limited. Furthermore, there are technical obstacles, since each protein behaves differently, or cannot retain its native state after the process of crystallization [53]. Therefore, the availability of different offline and online tools for 3D protein modeling is crucial for the prediction of the protein function. Furthermore, PPIs, as well as PTMs, play a central role in the regulation of a large number of cellular signaling processes, and alteration of the interactions and PTMs may lead to disease [54,55].

After modeling the wild-type and mutated DNAI1 and SPAG16 proteins, alteration of the native structure was seen in mutated DNAI1 protein harboring the p.Asn450LeufsTer6 genetic variant, as well as in SPAG16 protein harboring one homozygous genetic variant, p.Ser356Asn (Figure 2c and Figure 3b). In the 3D model of DNAI1 protein with p.Asp562Asn genetic variant, the aspartic acid is replaced with asparagine acid in the polypeptide chain; however, this amino acid change did not affect the tridimensional structure of the protein (Figure 2d).

When the ligand-binding sites and conserved domains were analyzed, results have shown that in DNAI1 protein harbors the information for premature stop codon (caused by p.Asn450LeufsTer6 genetic variant), the ligand-binding sites, and WD-40 domain are missing, thus implying that the protein lacks its biological function. The DNAI1 protein harboring the amino acid change (caused by p.Asp562Asn genetic variant) lost the ligand-binding site in position 562 due to the amino acid replacement. WD-40 domain is responsible for protein–protein interactions during the formation of large multiprotein complexes and is present in many eukaryotic proteins. Any change in the amino acid sequence within this domain affects the specificity of the protein interactions [56].

The ligand-binding sites, except one in position 356 and five detected WD-40 domains, were preserved in SPAG16 protein. Amino acid serine in 356 position in the polypeptide chain of the SPAG16 protein is replaced with amino acid asparagine (caused by p.Ser356Asn), which may affect the PPIs. Although the positions of ligand binding were preserved, the native structure of the protein was altered due to genetic changes, which could have led to the inability to access the ligands to the ligand-binding sites.

Applying the tool for detection of PTMs, in the DNAI1 protein, the phosphorylation and acetylation were detected, while in the SPAG16 protein phosphorylation, acetylation and ubiquitylation were detected. Phosphorylation of proteins is an important posttranslational modification since it acts as a molecular switch for many biological functions, such as activation and deactivation of some proteins, changing the conformation of the protein and others [57,58]. Acetylation increases the diversity and complexity of the cellular machinery and moreover regulates this machinery [59]. Ubiquitylation is a PTm that functions as a degradation mechanism for proteins and it has a role in DNA damage repair [60]. The analysis of the sequence conservation has shown complete evolutionary conservation among all analyzed species in both analyzed proteins.

Functional characterization within this pipeline included the measurements of the relative gene expression levels and immunoblotting of targeted proteins. The utilization of the gene-editing technology to further elucidate the influence of the detected variants on the phenotype is the last phase within the third step. This technology was not applied and validated within this study.

The results of the analyzed mRNA of the *DNAI1* gene that has both altered and premature stop codon showed that the level of expressed mRNA in patient P21 was reduced by approximately 50% compared to wild-type mRNA analyzed in the control group of subjects. Comparing the results of the *DNAI1* expression levels in patients harboring different stop mutations, research found that patients who have genetic variants c.947_948insG or c.1345_1349delCTTAA have approximately the same level of expression, while the level of expression compared to the control group of subjects decreased by around 50%, which leads to the conclusion that the mRNA that contains information for the premature stop codon is marked for degradation by nonsense-mediated mRNA decay [61]. Analysis of the DNAI1 protein containing both genetic variants p.Asn450LeufsTer6 and p.Asp562Asn revealed the presence of a protein of 699 amino acids, and a truncated protein of 455 amino acids was detected. On the basis of the obtained results, it can be assumed that the mRNA transcribed from the allele carrying the genetic variant c.1684G > A (p.Asp562Asn) is translated, while the mRNA transcribed from the other allele, with the genetic variant p.Asn450LeufsTer6, is marked for degradation. However, this process is not equally effective in all cell types, and consequently, a shorter protein product of 455 amino acids was detected.

## 4. Materials and Methods

Biological samples from two patients with PCD and family members were collected in collaboration with the Institute of Mother and Child Health Care “Dr. VukanCupic”. The study was conducted according to the guidelines of the Declaration of Helsinki and approved by the Ethics Committee of the Mother and Child Health Care Institute of Serbia “Dr. VukanCupic” (approval number: 01/1150/1, date: 31/01/2017).in Belgrade, Serbia. PCD was diagnosed in patients during neonatal screening, in childhood or adulthood, at the mentioned institute. Criteria for establishing the diagnosis of PCD included the following symptoms: neonatal respiratory distress at birth, frequent pneumonia, chronic sinusitis, chronic otitis media, bronchiectasis, and situs inversus. To determine the motility of motor cilia, we sampled airway epithelial cells and detected their motility using an optical microscope. The exclusion criteria for other lung diseases included testing patients for the presence of *P. aeruginosa* and performing a sweat chloride test to rule out cystic fibrosis (CF) as the final diagnosis.

For genomic profiling, the control group consisting of 70 subjects from the territory of Serbia was used. For the RT-qPCR analysis, 5 healthy controls corresponding to the patient by sex and age, as well as 2 confirmed PCD patients, were used. One control sample was used for the Western blot analysis.

The study included designing a pipeline for the detection and classification of the rare genetic variants detected using next-generation sequencing. The pipeline included both in silico and functional analysis of the detected variants.

The variant (previously reported) within the *SPAG16* gene (NM_024532.4) was analyzed, guided by this pipeline using an in silico approach. Using both in silico and functional analysis, we analyzed the two (previously reported) variants within the *DNAI1* gene (NM_012144.2).

### 4.1. Databases and In Silico Tools for Analysis of Detected Genetic Variants

The databases used to determine the potential pathogenicity of the detected variants in *DNAI1* gene are: OMIM (https://www.omim.org/ (accessed on 1 June 2021)), HGMD (http://www.hgmd.cf.ac.uk/ac/index.php (accessed on 1 June 2021)), InterVar (http://wintervar.wglab.org/ (accessed on 1 June 2021)), ClinVar (https://www.ncbi.nlm.nih.gov/clinvar/ (accessed on 1 June 2021)), VarSome (https://varsome.com/ (accessed on 1 June 2021)), SIFT (https://sift.bii.astar.edu.sg/ (accessed on 1 June 2021)), PROVEAN (http://provean.jcvi.org/ (accessed on 1 June 2021)), PolyPhen-2 (http://genetics.bwh.harvard.edu/pph2/ (accessed on 1 June 2021)), and Translate Tool software (https://web.expasy.org/cgibin/translate/ dna2aa.cgi (accessed on 1 June 2021))

In silico tools used for the creation of 3D structures, prediction of protein–ligand binding sites, protein–protein interactions, protein post-translational modifications, and evolutionarily conserved regions were: Phyre2 (http://www.sbg.bio.ic.ac.uk/ (accessed on 1 June 2021)), Raptor X(http://raptorx.uchicago.edu/predicts (accessed on 1 June 2021)), I-TASSER (https://zhanglab.dcmb.med.umich.edu/I-TASSER/ (accessed on 1 June 2021)), COACH (https://zhanglab.ccmb.med.umich.edu/COACH/ (accessed on 1 June 2021)), STRING (https://string-db.org/ (accessed on 1 June 2021)), PhosphoSitePlus (https://www.phosphosite.org (accessed on 1 June 2021)), and Aminode (http://www.aminode.org (accessed on 1 June 2021)). The modeled 3D structures were in PDB format (Protein Data Base, PDB), and for their analysis, the computer program UCSF Chimera 1.14 was used, which enables interactive visualization and analysis of molecular structures [62].

### 4.2. Functional Analysis of the Genetic Variants within the DNAI1 Gene

#### 4.2.1. Isolation of Genomic DNA

Genomic DNA was isolated from peripheral blood of patients and control subjects using a commercially available kit, which has a detection range from 0.2 to 100 ng DNA (QIAamp DNA Blood Mini Kit, QIAGEN, Hilden, Germany). Quantification of DNA molecules was performed using a Qubit 3.0 fluorimeter (Invitrogen, Carlsbad, CA, USA), according to the manufacturer’s instructions.

#### 4.2.2. Genomic Profiling

The study included genomic profiling of two patients suspected of PCD, as well as a control group consisting of 70 subjects from the territory of Serbia. All subjects were analyzed by next-generation sequencing technology. TruSight One Sequencing Panel (Illumina, San Diego, CA, USA) was used to prepare the library for sequencing. Sample preparation and formation of the DNA library were performed through applying Reagent Kit V3 (Illumina, San Diego, CA, USA)), and according to the manufacturer’s protocol (https://support.illumina.com/ (accessed on 20 March 2018)). After preparation of the library and its validation, the obtained concentration was converted to 12–13 pm in a final volume of 600 μL inserted into the cartridge. The sequencing reaction was performed by a MiSeq sequencing apparatus (Illumina, San Diego, CA, USA). The analysis of the obtained data was performed with the Illumina Variant Studio 3.0 software (Illumina, San Diego, CA, USA), which analyzes VCF (variant call format, VCF) files. After applying these filters and in-house PCD gene panel including 29 genes, in the way explained in our previous study [23], we obtained around 100 variants per sample.

The obtained results of NGS analysis were verified by sequencing the regions of interest of DNA molecules according to the Sanger method. The reaction was performed on an automatic sequencer 3130 Genetic Analyzer (Applied Biosystems, Waltham, MA, USA), while the obtained results were analyzed using Sequencing Analysis Software v5.3.2 with KB Basecallerv1.4 (Applied Biosystems, Waltham, MA, USA). A list of primers used for PCR reactions and Sanger sequencing reactions is given in Table 1.

#### 4.2.3. Isolation of Mononuclear Cells and RNA Molecules, and cDNA Synthesis

Mononuclear cells were isolated from biological samples (whole peripheral blood) of patients and controls using Ficoll-Paque™ Plus (GE Healthcare, Chicago, IL, USA) to separate the cells by centrifugation. Isolation of complete RNA molecules was performed using TRI Reagent Solution (Thermo Fisher Scientific, Waltham, MA, USA), according to the manufacturer’s protocol. The quality and quantity of the isolated RNA molecule were checked using a Qubit RNA BR assay and a Qubit 3.0 fluorimeter (Invitrogen, Carlsbad, CA, USA). The detection range of this assay was between 20 and 1000 ng/μL RNA.

Complementary DNA synthesis was enabled using RevertAid M-MuLV reverse transcriptase (Thermo Scientific, Waltham, MA, USA), a random hexamer primer, and 1 μg of the pre-isolated RNA molecule.

#### 4.2.4. Relative Quantification of the *DNAI1* Gene Expression

The measurement of the relative expression level of the *DNAI1* gene was conducted on one patient with genetic variants labeled as P21; 2 confirmed PCD patients labeled as P9 and P10, harboring frameshift stop mutation, c.947_948insG [23]; and 5 healthy controls corresponded to the patient by sex and age.

Determination of the *DNAI1* gene expression profile was performed on the device ABI 7500 Real-Time PCR System (KAPA Biosystems, Wilmington, MA, USA) using the KAPA SYBR Green Universal qPCR kit (KAPA Biosystems, Wilmington, MA, USA) as an intercalating agent. The glyceraldehyde 3-phosphate dehydrogenase (glyceraldehyde 3-phosphate dehydrogenase, *GAPDH*) gene was used to normalize the samples, and the median dCt value of healthy controls was taken as a calibrator. Relative quantification analysis was performed by a comparative ddCT method. All experiments were performed in duplicate. A list of primers used for gene quantification is given in Table 2.

#### 4.2.5. Isolation of Proteins

Patient and control blood samples were treated with cell lysis buffer in a ratio of 1:1; then, after a series of washings with PBS buffer (phosphate-buffered saline; 0.1 M sodium phosphate, 0.15 M NaCl, pH 7.2), we added Tris-EDTA-NaCl (TEN) with protease inhibitor (Roche, Basel, Switzerland). After centrifugation, 0.25 M Tris-HCl (pH 7.8) was added to the precipitate, followed by a series of three cycles of samples incubation at 37 °C and freezing in liquid nitrogen at −196 °C.

Quantification of protein was performed on TECAN (Infinite M200 Pro, Männedorf, Switzerland) using the Bradford assay (Bio-Rad, Hercules, CA, USA), according to the manufacturer’s protocol.

#### 4.2.6. Western Blot Analysis

A patient with two genetic variants was included in the analysis of the influence of the genetic variants on the structure and function of proteins, as well as a control sample from the general population of Serbia.

Western blot analysis was performed using an Anti-Dynein intermediate chain 1 antibody (Abcam (ab166912), Cambridge, UK), a primary rabbit monoclonal antibody, which is specific for DNAI1 protein. The incubation lasted overnight at a temperature of +4 °C. The primary antibody was diluted in the recommended ratio of 1:1000. Secondary antibody (anti-rabbit IgG antibody conjugated to horseradish peroxidase (GE Healthcare, Chicago, IL, USA) was diluted 1:10,000 and incubated for 1 h at room temperature. A chemiluminescent agent (GE Healthcare, Chicago, IL, USA) was used to visualize the antigen–antibody complex. Detection of formed complexes is enabled using the ChemiDoc MP Imaging System (Bio-Rad, Hercules, CA, UK).

## 5. Conclusions

Applying the designed pipeline, we achieved the important findings. The variants within the *DNAI1* gene, the well-known PCD disease-causing gene, and the homozygous variant within the *SPAG16* gene, the candidate gene for PCD, were characterized as pathogenic, which led to the confirmation of PCD diagnosis. Therefore, the pipeline contributed to the stronger association of the *SPAG16* gene with the PCD; hence, it is proposed to be included in the PCD diagnostic gene panel.

In summary, we demonstrated that the combination of different databases, tools, software, prediction platforms, and experiments reduced the list of genetic variants from about 9000 down to a few. The variants labeled as VUS gained diagnostic significance after additional experiments and enabled the possible shift from candidate to PCD disease-causing gene.

It has been shown that the usage of the designed pipeline is simple, time-saving, and helpful in finding the genetic cause in PCD patients. However, this approach needs to be applied in more studies in order to be validated. We believe that this approach could contribute to the basic knowledge of the genetic background of the PCD, which will inevitably lead to a more reliable PCD diagnosis.

## Figures and Tables

**Table 1:** List of primers used for sequencing analysis.

Name	Sequence	Orientation	Length
**DNAI1_F_ex14**	5′-CATGGGATTCTGGGAAATGGGC-3′	Forward	22
**DNAI1_R_ex14**	5′-CTGTAGCCACAGACAGAGGG-3′	Reverse	20
**DNAI1_F_ex17**	5′-GTGTGCTGAGGGTGGGAAG	Forward	19
**DNAI1_R_ex17**	5′-GAACACAGTAGAAGAGTATGGCGC-3′	Reverse	24

**Table 2:** Primers for the *DNAI1* gene quantification.

Name	Sequence	Orientation	Length
**DNAI1_F_Exp**	5′-GTCCCAAGCTGCTAAGATCATGGAGCG-3′	Forward	27
**DNAI1_R_Exp**	5′-CAGGGTACCCACCTGGTCCC-3′	Reverse	20

## Data Availability

The authors confirm that the data supporting the findings of this study are available within the article and its supplementary materials. GenBank accession numbers for the sequences used in the analyses were as follows: NM_012144.2 (*DNAI1*), NM_024532.4 (*SPAG16*).
